# Case report: Olanzapine-associated water retention, high blood pressure, and subsequent preterm preeclampsia

**DOI:** 10.3389/fpsyt.2023.1301348

**Published:** 2023-11-20

**Authors:** Julia Izsak, Dimitra Falari, Pia Arnbert, Daniel Pouragheli, Jenny M. Kindblom, Daina Lasaitiene

**Affiliations:** ^1^Department of Clinical Pharmacology, Sahlgrenska University Hospital, Gothenburg, Sweden; ^2^Institute of Neuroscience and Physiology, The Sahlgrenska Academy, University of Gothenburg, Gothenburg, Sweden; ^3^Department of Psychiatry, Sahlgrenska University Hospital, Gothenburg, Sweden; ^4^Institute of Medicine, The Sahlgrenska Academy, University of Gothenburg, Gothenburg, Sweden; ^5^Department of Rheumatology, Södra Älvsborg Hospital, Borås, Sweden

**Keywords:** olanzapine, preeclampsia, hypertension, water retention, pregnancy

## Abstract

Olanzapine is one of the most frequently used antipsychotic medications during pregnancy, but information about its safety and adverse effects profile during pregnancy is scarce. We herein describe a case of a pregnant woman with several psychiatric disorders who developed water retention, hypertension, and subsequent preterm preeclampsia 3 weeks after initiation of treatment with olanzapine. To the best of our knowledge, this is the first case of olanzapine-associated preeclampsia described in literature.

## Introduction

Psychiatric disorders are common among women of reproductive age, even during pregnancy (1). Worldwide, about 10% of pregnant women experience mental disorders according to estimation by the World Health Organization (2). Over the past decade, the use of second-generation antipsychotics, including olanzapine as a mood stabilizer, has increased rapidly among both the general population and pregnant women (3). Although the safety of antipsychotic medications in pregnancy is an area with significant gaps in evidence, observational studies suggest that olanzapine is safe with respect to congenital malformations (4, 5). Data related to pregnancy complications in relation to treatment with olanzapine are scarce except for two observational studies reporting an increased risk of gestational diabetes and the newborn being large for gestational age in females treated with olanzapine during pregnancy (6, 7). As for preeclampsia, while observational studies have been pointing toward a possible association between pharmacotherapy during pregnancy and development of preeclampsia, no previous report has described development of preeclampsia during treatment with olanzapine (8–10). With respect to the scarcity of literature related to psychotropic pharmacotherapy during pregnancy, it is of utmost importance to report experiences and adverse events for the improved care of this patient group.

Here, we present a case of a 32-year-old woman with depression and anxiety where introduction of olanzapine treatment during the 26th week of pregnancy was associated with onset of water retention, elevated blood pressure, subsequent preeclampsia, and preterm delivery in gestational week 29.

## Case presentation

A 32-year-old Caucasian pregnant woman with a history of generalized anxiety disorder, emotionally unstable personality disorder, obsessive-compulsive disorder, posttraumatic stress disorder as well as one previous suicide attempt several years ago, was admitted to the maternity ward in gestational week 24 due to severe hyperemesis gravidarum, suicidal ideation, and increased anxiety. She had no family history of preeclampsia and she had stopped smoking during pregnancy.

The patient had been mentally stable for several years on treatment with lamotrigine 150 mg/day and venlafaxine 225 mg/day. A gradual deterioration of her mental health began after the sudden death of her mother 6 months prior to the current event. Around the same time, the patient discovered that she was pregnant. Her primary care physician began tapering her lamotrigine and venlafaxine, after consulting a psychiatrist. Her medications at the time of admission to the maternity ward were lamotrigine 25 mg daily and venlafaxine 187.5 mg daily. She was transferred to the psychiatry ward 6 days after being admitted to the maternity ward. At admission to the psychiatric ward (the 25th week of pregnancy), the patient’s weight was recorded at 85 kg. The patient’s self-recorded weight pre-pregnancy was approximately 73 kg (BMI 25.9). At the time of admission, her vital signs included a blood pressure of 105/59 mm Hg, a heart rate of 88 beats/min, and an oxygen saturation of 97%. Laboratory tests were normal except for lower serum albumin measuring 28–27 g/L and positive urine protein ranging from 1 to 2 + which is equivalent to 0.3–1 g/L protein in the urine. Positive urine protein finding had been present as early as gestational week 10 when the patient sought medical attention for hyperemesis gravidarum. Of note, during her pregnancy, there were intermittent instances of negative urine protein results. Her hypoalbuminemia and proteinuria were not evaluated further during her hospitalization.

**Figure 1:**
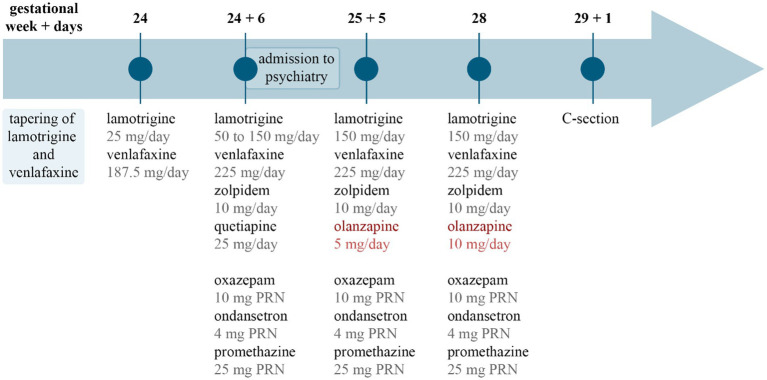
Timeline of drug treatment. The image summarizes drug treatment changes over time. Gestational week and days are marked at the top, and pharmacotherapy is listed below. Circles and lines in the middle mark the time points related to each drug list. PRN, *pro re nata*, as needed; C-section, cesarean section.

During the patient’s hospitalization, her psychotropic treatment was intensified with the gradual increase of lamotrigine to 150 mg/day over 2 weeks and increase of venlafaxine to 225 mg/day. Following dose increase, plasma levels of venlafaxine and its active metabolite were within the therapeutic range. In response to significant sleep disturbances in the beginning of hospitalization, a nightly regimen of 10 mg zolpidem and 25 mg quetiapine was initiated. She additionally received oxazepam 10 mg and promethazine 25 mg for anxiety as well as ondansetron 4 mg for nausea as needed (*pro re nata*: PRN). The restoration of her sleep quality prompted the discontinuation of quetiapine after a total of 10 days of use. Concurrently, the patient’s clinical picture was dominated by severe anxiety with intermittent suicidal thoughts that led to the introduction of olanzapine, 2.5 mg twice a day (BID), with subsequent gradual increase to 7.5 mg/day and then 5 mg BID during the following 2 weeks. She was treated at the psychiatry ward for a duration of 25 days, during which she received olanzapine for 20 days. She was discharged with scheduled outpatient psychiatric follow-up, including planned monthly concentration measurements of lamotrigine with potential dose increase if required. For a summarized overview of the drug treatment regimen over the course of the disease, see Figure 1.

A retrospective evaluation showed that the body weight increased 16.5 kg during the 3-week period following the introduction of olanzapine. Significantly, 14 kg of this weight gain occurred within the last 2 weeks of this period. Blood pressure measurements were sporadically recorded with readings in the range of 105/59–111/73 mm Hg before, and 150/92 mm Hg at discharge, 3 weeks after the introduction of olanzapine.

The patient was readmitted to the hospital 2 days after her discharge, at gestational week 28 + 6 with dyspnoea, extreme weight gain, and high blood pressure measuring 163/114 mm Hg. Her heart rate was recorded at 98 beats/min and oxygen saturation was at 95%. Additionally, peripheral oedema was observed in her lower legs and hands. Extensive laboratory testing, with only abnormal results reported revealed low hemoglobin level, ranging between 105 and 107 g/L, low serum albumin measuring 19–20 g/L, increased NT-proBNP—2,410 ng/L, and high urine albumin to creatinine ratio of 1,340 g/mol (reference <5 g/mol). Blood sugar, HbA1C, screening for viral infections, and thrombophilia were normal. Chest computer tomography revealed bilateral pleural effusion but showed no signs of pulmonary embolism. Echocardiography revealed signs of fluid overload, with all four chambers slightly dilated, but the heart function was within the normal range. The patient was prescribed labetalol, clonidine, nifedipine, and furosemide for hypertension and oedema as well as magnesium sulfate for seizure prophylaxis. An urgent cesarean section was performed 2 days later, at gestational week 29 + 1, due to preeclampsia. The newborn’s birth weight was 1,200 g, and the Apgar scores at 1, 5, and 10 min were 8, 7, and 7, respectively. No growth restriction or developmental abnormalities were noted at birth. Postpartum microscopic pathological evaluation of the placenta was consistent with preeclampsia, where accelerated maturation and hypoxic changes as well as microinfarctions were present.

The symptoms of dyspnoea and peripheral oedema, as well as serum albumin and NT-proBNP started to improve promptly postpartum. Her blood pressure was controlled by daily doses of 600 mg labetalol, 5 mg enalapril, and 40 mg furosemide, and subsequent echocardiography revealed no pathological changes. The patient was mentally stable and was transferred to neonatal intensive care unit 5 days postpartum, for medical care of her newborn. Psychiatric medication remained unchanged at discharge, i.e., lamotrigine 150 mg/day, venlafaxine 225 mg/day, zolpidem 10 mg/day, olanzapine 10 mg/day, oxazepam 10 mg PRN, and promethazine 25 mg PRN.

## Discussion

Preeclampsia is a life-threatening complex multisystem disease that can occur during pregnancy, characterized by onset of elevated blood pressure and often accompanied by complications such as proteinuria, organ dysfunction, or placental dysfunction. The pathophysiological mechanisms of preeclampsia are not completely understood but certain risk factors have been suggested to contribute to the condition, such as nulliparity, chronic hypertension, pregestational diabetes, family history of preeclampsia, high BMI, and maternal age (11).

### Preeclampsia and olanzapine

The etiology of preeclampsia is not fully understood. Observational studies have suggested a possible association between pharmacotherapy during pregnancy and the development of preeclampsia, but no definitive causal relationship has been described (8–10). No previous report has described preeclampsia in association with olanzapine treatment. Interestingly, *in vitro* experiments on human trophoblast cells exposed to olanzapine reported altered trophoblast invasion, a phenomenon that is implicated in adverse pregnancy outcomes, such as preeclampsia (12), but it is unknown whether this *in vitro* observation has relevance in a clinical context.

Hypertension and peripheral oedema are known side effects of olanzapine, with a reported incidence of 2–3% in the general population (13). The mechanism behind olanzapine-induced hypertension and oedema is not well understood, but several somewhat contradictory mechanisms have been suggested. For instance, hypertension has been discussed to be related to renal dopamine receptors where antagonism by olanzapine may trigger an increase in blood pressure by interacting with the renin-angiotensin-aldosterone system (14, 15). Further, dopaminergic antagonism might also play a role in the development of oedema as hypodopaminergic states have been described to be associated with disrupted fluid and electrolyte balance as well as idiopathic oedema (16). Olanzapine-associated oedema has been discussed to be related to a line of additional mechanisms, converging to a peripheral vasodilating effect. One such mechanism is alpha adrenergic receptor blockade resulting in vasodilatation and decreased vascular resistance (17–19). Further, antagonism of histaminergic and serotonergic receptors by olanzapine causing a subsequent downregulation of ATP-dependent calcium pump, has also been suggested as a potential mechanism leading to vascular muscle relaxation (17–19). Serotonin receptor blockade has been even proposed to potentially increase cAMP, causing vascular smooth muscle relaxation by phosphorylation of myosin light chain kinase (17–19). Yet, preeclampsia has not been described previously as an adverse effect of olanzapine. Interestingly, for aripiprazole, a similar atypical antipsychotic medication, an increased risk of hypertension in pregnancy and of preterm birth has been reported (20), although without observations of increased risk of preeclampsia (21). Olanzapine use during pregnancy is known to be associated with an increased risk of gestational diabetes (22), but in our case, diabetes was not present.

In the current case, the timeline of events with water retention, high blood pressure, and preeclampsia culminating 22 days after initiation of olanzapine treatment suggests a possible causal relationship.

However, other factors, which could have influenced development of hypertension and preeclampsia, cannot be disregarded. The underlying depression and anxiety may have contributed to an increased risk of developing high blood pressure and preeclampsia (23, 24). Further, hyperemesis gravidarum, and especially second-trimester hyperemesis, has also been linked to an increased risk of preeclampsia (25). Another known factor that can be mentioned in the current case is the presence of proteinuria, which has been described as an important predictor for developing preeclampsia (26). Assessment of the adverse drug reaction using the Naranjo adverse drug reaction probability score gives one point for the side effect of preeclampsia and two points for hypertension, and the interpretation of “possible” adverse drug reaction (27). Due to the patient’s clinical course, the answers related to discontinuation and rechallenge remain unanswered.

### Psychiatric medication and polypharmacy during pregnancy

In general, for the treatment of psychiatric diseases during pregnancy, a central critical question is whether the benefits of pharmacological interventions outweigh the harms for woman and child. The available evidence does not clarify this issue. Nevertheless, current guidelines and clinical practice suggest that treatment should be continued when there is a high-assessed risk of relapse or recurrence of psychiatric disorder (28). In this specific case, the fact that the patient was stable for a long time led to tapering during pregnancy. Yet, given the occurrence of a serious life event, the death of her mother, tapering psychiatric medication is associated with a high risk of relapse. Tapering of antidepressant treatment in conjunction with the discovered pregnancy and subsequent addition of the second-generation antipsychotic medication after clinical worsening, might have contributed to the development of preeclampsia and emergency cesarean section.

The patient was further treated with a long list of medicines: lamotrigine, venlafaxine, oxazepam, quetiapine, ondansetron, promethazine, and zolpidem, which complicates assessment of a possible causal relationship between olanzapine and preeclampsia. No pharmacokinetic interactions have been found between the patient’s medicines and olanzapine, thus we do not expect a higher exposure to olanzapine in this case (29). Interestingly, hypertension and oedema are described even as possible side effects of venlafaxine, especially at daily doses over 300 mg (30, 31). Although our patient was taking a lower dose of venlafaxine for years, an additive pharmacodynamic effect with olanzapine cannot be ruled out.

### Retrospective analysis of clinical course and decisions

As mentioned earlier, tapering antidepressant medication in early pregnancy, in a period with a serious life event, increases the risk of relapse. Further, at week 10, when the patient sought medical help due to hyperemesis, worsening of her mental health could have been recognized and her medication could have been increased to prevent further deterioration.

After hospital admission at gestational week 24, the patient was prescribed quetiapine 25 mg as off-label treatment for sleep. As sleep quality improved, the medication was discontinued after a total of 10 days. An adjunctive therapy with olanzapine was started at the same time by reason of the dominance of severe anxiety with intermittent suicidal thoughts in the clinical picture. At this point, instead of introducing olanzapine, another alternative would have been to increase the quetiapine dose, thereby minimizing exposure to new medications during pregnancy. In the context of severe and acute deterioration of the patient’s symptoms, our decision to augment with olanzapine was driven by the potentially lengthy process of up-titrating quetiapine, coupled with its poor tolerability (32). Olanzapine’s effectiveness at lower doses in augmenting anxiety treatment was another significant factor in our decision to change medication (33). Moreover, we have substantial experience and a longstanding tradition in Sweden of the use of olanzapine as mood stabilizer during pregnancy.

An additional important aspect to mention is the alarming weight gain of over 16 kg in 3 weeks, considering the increased risk of diabetes, metabolic disorders, hypertension, and the infant being large for gestational age (34). Yet, the patient did not develop diabetes and the baby was born with normal weight for gestational age. Given the rapid weight increase within such a brief timeframe, it is likely that this gain in weight was due to a combination of water retention and actual weight gain.

Lastly, hypoalbuminemia and proteinuria, along with their quantification, could have been considered and followed up with additional checks, as they can be important predictors for preeclampsia (26).

Taken together, several risk factors including psychiatric comorbidity, hyperemesis gravidarum, higher BMI, proteinuria, polypharmacy, and treatment changes might have contributed to the risk of developing water retention, elevated blood pressure, and eventually preeclampsia in our case. Yet, the striking temporal relationship between initiation of olanzapine treatment and escalation of symptoms suggests that olanzapine-induced water retention and hypertension, known side effects of this compound, may have contributed to the development of preterm preeclampsia and preterm cesarean section in our case.

## Conclusion

We hereby present the first case report of preeclampsia with preterm birth, preceded by water retention and hypertension during pregnancy, and a striking temporal relationship with olanzapine treatment. This case highlights the importance of enhanced monitoring of body weight, blood pressure, and urine protein in patients on pharmacotherapy during pregnancy. More research is needed to understand whether olanzapine contributes to the development of preeclampsia and to ensure safe pharmacotherapy for women and children.

## Data availability statement

The original contributions presented in the study are included in the article/supplementary material, further inquiries can be directed to the corresponding author.

## Ethics statement

Written informed consent was obtained from the individual(s) for the publication of any potentially identifiable images or data included in this article.

## Author contributions

JI: Conceptualization, Data curation, Investigation, Methodology, Writing – original draft, Writing – review & editing. DF: Writing – review & editing. PA: Writing – review & editing. DP: Writing – review & editing. JMK: Writing – review & editing. DL: Conceptualization, Data curation, Investigation, Methodology, Supervision, Writing – original draft, Writing – review & editing.
